# Liver Bioreactor Design Issues of Fluid Flow and Zonation, Fibrosis, and Mechanics: A Computational Perspective

**DOI:** 10.3390/jfb11010013

**Published:** 2020-02-28

**Authors:** Vahid Rezania, Dennis Coombe, Jack Tuszynski

**Affiliations:** 1 Department of Physical Sciences, MacEwan University, Edmonton, AB T5J 4S2, Canada; rezaniav@macewan.ca; 2 Computer Modelling Group Ltd., Calgary, AB T2L 2A6, Canada; Dennis.Coombe@cmgl.ca; 3 Department of Physics and Experimental Oncology, University of Alberta, Edmonton, AB T6G 2J1, Canada; 4 DIMEAS, Politecnico di Torino, Corso Duca degli Abruzzi 24, 10129 Torino TO, Italy

**Keywords:** virtual liver, multi-scale modeling, lobule, tissue engineering, zonation, fibrosis, upscaling, drug transport, dual continuum modeling

## Abstract

Tissue engineering, with the goal of repairing or replacing damaged tissue and organs, has continued to make dramatic science-based advances since its origins in the late 1980’s and early 1990’s. Such advances are always multi-disciplinary in nature, from basic biology and chemistry through physics and mathematics to various engineering and computer fields. This review will focus its attention on two topics critical for tissue engineering liver development: (a) fluid flow, zonation, and drug screening, and (b) biomechanics, tissue stiffness, and fibrosis, all within the context of 3D structures. First, a general overview of various bioreactor designs developed to investigate fluid transport and tissue biomechanics is given. This includes a mention of computational fluid dynamic methods used to optimize and validate these designs. Thereafter, the perspective provided by computer simulations of flow, reactive transport, and biomechanics responses at the scale of the liver lobule and liver tissue is outlined, in addition to how bioreactor-measured properties can be utilized in these models. Here, the fundamental issues of tortuosity and upscaling are highlighted, as well as the role of disease and fibrosis in these issues. Some idealized simulations of the effects of fibrosis on lobule drug transport and mechanics responses are provided to further illustrate these concepts. This review concludes with an outline of some practical applications of tissue engineering advances and how efficient computational upscaling techniques, such as dual continuum modeling, might be used to quantify the transition of bioreactor results to the full liver scale.

## 1. Introduction

Tissue engineering, with the goal of repairing or replacing damaged tissue and organs, has continued to make dramatic science-based advances since its origins in the late 1980’s and early 1990’s. Such advances are always multi-disciplinary in nature, from basic biology and chemistry through physics and mathematics to various engineering and computer fields.

Numerous textbooks on tissue engineering provide a general overview and historical perspective of the field. This includes the comprehensive book of Lanza et al. [1], as well as the more process analysis-based textbook of Palsson and Bhatia [2]. Chaudhuri and Al-Rubeai [3] have provided an analysis on various bioreactor designs, whilst Sharma [4] has given an in-depth treatment of basic computational concepts. Furthermore, Burdick and Mauck [5] have given on overview of the biomaterials used in tissue engineering. A large number of review articles provide updates on recent advances in many interrelated areas. These will be highlighted throughout our review article.

Many of the factors important for successful tissue engineering have now been agreed upon for tissue culture success. These include the advantages of the co-culturing of cell types versus single cell only cultures; the use of 3D versus 2D culture methods and scaffolding; the impact of fluid flow, fluid shear stress, and nutrient (especially O_2_) transport for cell culture maintenance; and the roles of extracellular matrices (ECM) and tissue stiffness in cell differentiation and growth. Basic data have been obtained via high-throughput microarrays, soft lithography and microfluidics, biomaterial scaffolding, and micro-contact printing and bioprinting methods. 

Ebrahimkhani et al. [6] have reviewed and classified the types of bioreactor technologies available, while Gural et al. [7] have discussed their utility in the study of infectious disease. These references provide an overview of modern liver culturing methods, including high throughput cell microarrays, micropatterned co-cultures, liver spheroids and organoids, and some microfluidic perfusion devices such as “liver-on-a-chip”. The use of bioreactor technology for the study of drug metabolism and toxicity has been emphasized in several recent reviews by two groups—Lin and Khetani [8], Underhill and Khetani [9,10], and Bale et al. [11]. Jain et al. [12] and Ye et al. [13] have summarized appropriate scaffolding choices for liver engineering studies involving both natural (e.g., collagen, alginate, and Matrigel) and synthetic (e.g., polyethelene-glycol and polycaprolactone) polymers that can be used for liver tissue bioengineering (see especially the tables these references provide).

Liver cell lines such as primary human hepatocytes (PHH) or human hepatocellular carcinoma (HepG2) are the most ideal for obtaining quantitative information, while rat hepatocytes or porcine hepatocytes are often used for research or bioartificial liver (BAL) applications, due to their cost and availability. Co-culturing with non-parenchymal cells such as liver sinusoidal endothelial cells (LSEC), as well as Kupffer cells or stellate cells, is recommended for extending culture viability and more mechanistic investigations of inflammation, respectively. The primary CYP enzyme of interest is CYP3A4/5, responsible for 50% of drugs metabolized, while CYP2D6 and CYP2B6 can metabolize about 25% drugs as well.

The first design issue is appropriate cell seeding on the chosen scaffolding, with uniform, high-density cell distributions desired. An appropriate choice of ECM is important (biodegradability, mechanical strength, immunogenicity, etc.) and the co-culturing of hepatocytes with other nonparenchymal cell types leads to improved and longer-lasting hepatocyte functioning. 

More specifically, other reviewers have discussed the roles of such factors and methods as applied to liver tissue engineering advances and applications. Hewitt et al. [14], and later Godoy et al. [15], provided extensive summaries of hepatocyte culturing methods, and the important role of the underlying and overlying matrix (e.g., collagen and Matrigel sandwich culturing method) for proper hepatocyte morphology and metabolite enzyme (cytochrome p or CYP450) expression. Berthiaume et al. [16] emphasized the role of ECM topology in cell morphology and function by emphasizing the changes induced in a sandwich culture system. It is worth noting that the essentials and variety of basic bioreactor design have been discussed in detail in a number of review articles, which are summarized in Section 2.

The use of various micro-engineered liver constructs for drug testing and drug toxicity has been reviewed by several authors. Yoon No et al. [17] present an excellent review of 3D microfluidic liver models and liver-on-a-chip. Khetani et al. [18] give an extensive review of micro-model constructs used in exploring drug-induced liver injury (DILI). Materne et al. [19] emphasize the tradeoffs required between cost-effective high throughput analysis and tissue-representative microfluidic design. Bale et al. [11] extend these considerations to consider inter-organ interactions, while Vishwakarma et al. [20] emphasize the role of a complete 3D architecture through the use of decellularized scaffolds with repopulation techniques in interpreting drug behavior.

While one focus can be on such advanced experimental methods, it has been proven equally important to place such experiments in a consistent mathematical framework and context, in terms of fundamental biochemical and biophysical mechanisms. Here, we wish to emphasize the coupling of such technology to computational methods to quantify and generalize such drug development results to more physiologically-relevant scenarios at multiple scales. Therefore, for the purposes of this review, we will assume hereafter that basic culture issues have been resolved in some manner. This is not to imply that there are no significant unresolved issues to be addressed in the future. Instead, we will focus our attention on two topics critical for tissue engineering liver development: (a) fluid flow, zonation, and drug screening, and (b) biomechanics, tissue stiffness, and fibrosis, all within the context of 3D structures. Thereafter, we will address (c) computational methods and upscaling issues. 

## 2. Fluid Flow, Zonation, and Drug Screening

### 2.1. Liver Lobule Characteristics and Zonation

In-vivo, the functional unit of the liver is the liver lobule at a scale of approximately 1 mm^3^ [21,22]. Volumetrically, the liver lobule consists of 84% cellular space (78% hepatocytes, 3% sinusoid endothelial cells, 2% Kupffer cells, and 1% stellate (Ito) cells) and 16% extracellular space (11% sinusoid lumina and 5% space of Disse). In terms of cell numbers, the same cell types represent approximately 64%, 16%, 12%, and 8%, respectively [19]. These are general characteristics that tissue engineering constructs should attempt to mimic.

The zonation (a non-uniform spatial distribution) of many metabolic processes within the lobule is what determines its functionality [23,24]. Such processes include carbohydrate, ammonia, lipid, and drug metabolism [25,26,27,28]. Zonation in itself is basically a consequence of O_2_ availability and its limitation is due to the high metabolic activity of hepatocytes [29,30,31,32]. The main motivator of liver tissue engineering design is therefore to mimic such zonation in-vitro, which primarily involves control of the O_2_ distribution.

Fundamental to all experimental tissue engineering design and computational aspects of liver modeling are the high metabolic activity of hepatocytes and their consequent high utilization/consumption of O_2_, which is approximately 10 times that of other cell types [33].

### 2.2. Basic Bioreactor Configurations and Analysis (Spheroids, Scaffolding, and Transport Issues)

The variety of basic bioreactor designs and their essential issues have been discussed in detail in numerous excellent review articles, which are summarized in this section. A wide variety of 2D and 3D scaffold fabrications have been investigated with the idea of generating, maintaining, and quantifying liver cell zonation. Traditional bioreactor models began with stirred suspensions and 2D flat-plate designs. Later 3D systems included spheroids, sandwich polymer systems, and porous or hydrogel encapsulation. Of these, only spheroids do not utilize an ECM cell encapsulation method. 

Three-dimensional liver spheroids (typically 200 µm diameter) exhibit improved hepatocyte activity over 2D culture systems, and have also been used to characterize liver disease and drug testing [34]. The spontaneous aggregation of these scaffold-free multi-cell-type systems, cultured in 96-well plates, generates their own extracellular matrix over time.

Swedish researchers have investigated a 3D hepatocyte (PHH) spheroid protocol (size of approximately 200 µm diameters, with 1500 viable cells) and used this to characterize liver function and disease, drug-induced toxicity, steatosis, and insulin resistance, as allowed by the long-term viability of their system [35,36].

However, this can result in the development of a necrotic inner zone as spheroids increase in size due to oxygen and nutrient diffusion limitations, even though they are originally scaffold-free systems. Therefore, perfusion microfluidic systems have been designed for enhanced transport around spheroids while minimizing shear stress. Lee et al. [37] developed and modeled a spheroid-based microfluidic liver-on-a-chip system, co-cultured with hepatocytes and stellate cells, to probe liver viability by monitoring albumin and urea secretion and CYP450 activity. They later utilized this experimental system to investigate alcoholic liver disease and recovery [38]. 

Two additional designs, which constrain the spheroids in a flow device, were optimized based on computational methods to minimize shear stress and have also been reported. Kim et al. [39] used their system to explore tumor spheroid growth, and Yu et al. [40] focused on O_2_ availability and drug (acetaminophen and diclofenac) toxicity, based on their previous design [41]. Detailed numerical simulations of flow-based spheroids embedded in hydrogels were presented by Sharifi et al. [42] and demonstrate the insight that such modeling provides for system optimization.

Sandwich culture systems have also been explored extensively. These systems typically use collagen-coated membranes to isolate cells from direct perfusion shear stresses while allowing for mass transfer effects to maintain cell viability. Early work by Dunn et al. [43] and Rotem et al. [44] emphasized the role of dual collagen layers in generating proper hepatocyte polarity and longer-term cell functionality. This was later upscaled by De Bartolo et al. [45]. Xia et al. [46] used a somewhat different sandwich design to investigate drug hepatotoxicity induced by acetaminophen (APAP).

Starting from initial bioreactor designs, computational modeling has been utilized to understand and control the O_2_ distribution, both for radial [47] and flat-plate bioreactors [48,49,50,51], as O_2_ availability for proper hepatocyte functioning is always a primary concern. 

Yarmush and coworkers have investigated several microfluidic designs, including 2D biochips with fluid recycling, to explore hepatic clearance and metabolite generation [52,53], which is based on a patented HuREL design with four biochips in parallel. They also utilized a flat-plate sandwich configuration with hepatocytes on collagen separated by a porous membrane from a flow compartment to investigate flow effects on hepatocyte viability [54]. More recently, a newer (Christmas tree inlet) design allowed a direct investigation of induced zonation effects by mixing five ports of varying inlet concentrations of inducing chemicals [55,56]. Here, the zonation of carbohydrate metabolism and nitrogen metabolism, alcohol degradation zonation, and drug conjugation zonation (for acetaminophen) were probed. Bhatia and coworkers used plate patterning to generate hepatocyte co-cultures that were then encapsulated into hydrogel microtissues (100 µm diameter). These were then utilized in microfluidic devices to investigate fluid flow effects on hepatocyte viability [57], and eventually on the induced pluripotent stem (iPS) liver cell differentiation [58].

The Griffith laboratory has fabricated and utilized variations of a 3D perfused bioreactor with cross flow through cell-populated co-cultured slits, ensuring sufficient O_2_ availability, but maintaining low fluid shear stress. Their initial bioreactor designs were outlined with considerations of O_2_ availability and hepatocyte functional activity [59,60]. This was later extended to co-culture effects and drug metabolism studies [61,62].

In particular, Buck et al. [63] measured the effects of the O_2_ concentration in the pericellular microenvironment of hepatocytes and the role of the extracellular matrix (Matrigel) on cell viability. Griffith and Swartz [64] emphasized the design principles necessary for the realistic capturing of complex 3D physiology when building in-vitro representations of tissue behavior. Later, extensive work on liver drug clearance and toxicity by Griffith’s group [65,66,67,68] employed LiverChip, a commercially available extension of this design.

Ahluwalia and coworkers [69] first modeled (via computational fluid dynamics (CFD)) and then developed a low shear stress modular bioreactor chamber system (termed MCmB) and applied this to hepatocyte cell systems. Here, the shear stress experienced by an ECM-coated cell system can be adjusted by varying the height of the cell coverslip from the flow system near the top of the bioreactor. This system was later extended to the study of liver glucose and lipid metabolism [70] and thereafter to liver detoxification by monitoring the up-regulation of liver xenobiotic metabolism genes [71]. Interestingly, they found that some CYP genes showed an increased expression in flow (e.g., CYP1A1), while others were unaffected (e.g., CYP2D6). Pedersen et al. [72] provided a computational analysis of this bioreactor flow system and compared it to two other bioreactor designs. More recently, Tomlinson et al. [73] used mathematical modeling and experiments to study liver zonation induced by a paracetamol injection with a commercially available system developed by Sbrana and Ahluwalia [74] that is similar to the previous setup.

### 2.3. Specialized Bioreactor Configurations (Sinusoid-Like Structures, Lab-on-a-Chip, Bioink and Rapid Prototyping, and Precision-Cut Liver Slices)

Investigators from other laboratories have developed a wide variety of microfluidic bioreactors to investigate aspects of the hepatocyte phenotype and functions. Illa et al. [75] developed a two-plate co-culture bioreactor (Exoliver) which utilizes a membrane to separate a flow region (high shear stress) from a non-flow region (low shear stress) containing hepatocytes. This was later enhanced to monitor the O_2_ distribution and zonation, by Moya et al. [76], and drug hepatotoxicity, by Ortega-Ribera et al. [77].

Kang et al. [78] mimicked the sinusoid structure by co-culturing hepatocytes and endothelial cells on opposite sides of a microporous membrane in commercial 6-transwell culture dishes and probed CYP2E1 activity over 48 days. They then ported this structure to Polydimethylsiloxane (PDMS)-constructed microfluidic devices with either single-channel or dual-channel configurations [79]. Du et al. [80] co-cultured murine cells of various types in a two-fluid chamber microfluidic chip with the chambers separated by a porous membrane, and analyzed the flow patterns computationally while confirming CYP450 metabolism and even neutrophil recruitment with lipopolysaccharide stimulation. Liu et al. [81] investigated a three-layer microfluidic system with the middle cell-containing layer subdivided into 16 chambers separated by micro-channels while the medium was recycled from the upper chamber through the middle to the lower chamber. With this system, they experimentally and computationally investigated the flow of nutrients and the metabolism and detoxification action of the liver cells.

Originally, Lee and coworkers simulated and developed [82,83] a circular microfluidic chamber with connected perfusion ports and channels to study aspects of cancer cell flow cultures. Thereafter, they developed an alternate ingenious microfluidic bioreactor that resembles a sinusoid structure, with a grid of closely spaced micro-channels acting as an endothelial-like barrier separating a cell culture chamber of hepatocytes from a fluid flow channel, constructed in a U-shaped form [84,85]. Several years later, Gori et al. [86] used a similar system to probe aspects of non-alcoholic liver disease.

Microfluidic organoids for drug screening (MODS) have been developed by Au et al. [87] based on 3D hydrogel micro-tissues and used to study hepatic co-culture systems. Liver functionality was tested via monitoring albumin secretion and CYP3A4 activities, and drug screening via the effects of acetaminophen on cell apoptosis and necrosis. Toh et al. [88] developed a 3D hepatocyte chip (3D HepaTox) with eight parallel cell-seeded channels separated by micro-pillars coupled to a linear concentration gradient generator as the input to the eight channels. This configuration was used to assess the drug toxicity at eight different concentrations for five hepatotoxic drugs. Yu et al. [89] provided a recent review of organoid-on-a-chip models for many cell tissue types, including brain, intestine, and liver systems.

Lee et al. [90] fabricated a highly bioactive ink for printing 3D porous structures containing collagen/alginate ECM and loaded with hepatogenic adipose tissue stem cells. The result is a regular porous structure of about 350 mm^3^ in size, with struts and pores separated by roughly 500 µm. They later extended these concepts [91] by directly using a liver decellularized extracellular matrix as bioink.

Bioprinted liver tissue has been constructed [92] by combining separate bioink of parenchymal (hepatocytes) and nonparenchymal (endothelial) cells and printing these with continuous deposition onto membranes with hepatocytes in the middle surrounded by endothelial cells to form a tissue with a thickness of 500 µm. These were cultured for several days and allowed to coalesce into liver tissue for studies of drug-induced toxicity. This same technique was also applied to the study of various compound-induced fibroses [93] and further explored by focusing on the role of Kupffer cells [94]. Ma et al. [95] developed a 3D hydrogel-based hepatic bioprinting system using human iPSC stem cells to explore the liver tissue maturation process. Huang et al. [96] emphasized the role CFD computational methods can play in microfluidic chip design and validation.

Kizawa et al. [97], utilizing the Regenova apparatus, first constructed liver spheroids and placed them on a 9 × 9 array of needles for culturing in a perfusion chamber, which resulted in a coalesced cubical tissue. This tissue displayed both sinusoid and bile duct features and allowed the testing of liver activities, such as glucose production and bile acid secretion.

The use of precision-cut liver slices has the advantage of maintaining the multicellular architecture of human liver tissue. These are typically obtained by coring 0.5 to 1 cm diameter cylinders of a donor liver, followed by slicing sections of <250 µm. This approach has been pioneered by Groothuis and coworkers [98,99,100], who developed an innovative microfluidic system to perfuse the resulting samples. This was furthermore coupled to online HPLC monitoring to analyze metabolism and liver drug responses [101]. This system was also applied to inter-organ interactions by utilizing slices of liver and intestine tissue in series [98]. Hadi et al. [102] further explored drug-induced liver metabolism and toxicity.

Mazza et al. [103] utilized decellularized human liver tissue cubes (5 mm^3^) as scaffolding and repopulated them with various hepatic cell types as a basis for transplant studies. Other research groups have utilized this type of technology to further investigate liver disease, e.g., Vatakuti et al. [104] and Paish et al. [105]. Work in this area has been summarized by Palma et al. [106] in a recent review.

### 2.4. Bioreactor Drug Testing

The use of various micro-engineered liver constructs for drug testing and drug toxicity has been reviewed by several authors. Yoon No et al. [17] have presented an excellent review of 3D microfluidic liver models and liver-on-a-chip. Khetani et al. [18] have given an extensive review of micro-model constructs used in exploring drug-induced liver injury (DILI). Materne et al. [19] emphasized the tradeoffs required between cost-effective high throughput analysis and tissue-representative microfluidic design. Bale et al. [11] extended these considerations to consider inter-organ interactions, while Vishwakarma et al. [20] emphasized the role of a complete 3D architecture through the use of decellularized scaffolds with repopulation techniques in interpreting drug behavior.

### 2.5. Summary

With the exception of precision-cut liver slices and some rapid prototype constructs, most flow bioreactor systems are characterized by simple, well-defined flow paths. Consequently, numerical simulations of such systems have typically and profitably employed very exact CFD methods. However, in-vivo systems at the scale of the liver lobule or larger, require by necessity averaging methods to characterize their flow patterns. Later sections of this review will emphasize such methods.

## 3. Tissue Stiffness and Biomechanics, Fibrosis, Steatosis, and Cancer

### 3.1. Basic Concepts: Cells, ECM, and Fundamental Mechanics Responses

In addition to the role of fluid flow in the maintenance of healthy liver tissue activity, an appropriate tissue matrix stiffness (deformation resistance) contributes to a normal liver cell function [107,108]. Furthermore, cell mechanosensors and mechanotransduction imply that these two factors are coupled. Various liver diseases (steatosis, fibrosis, cirrhosis, and cancer) represent altered states of tissue stiffness. 

The mechanical response of liver tissue has both cellular and surrounding matrix (ECM) contributions, as cells adhere to ECM through specific focal adhesion sites via transmembrane integrins. Lim et al. [109] and Janmey and McCulloch [110] reviewed various viscoelastic modeling approaches for individual cells. At a macroscopic level, the studies by Kerdok [111] and Moran [112] give overviews of liver tissue (cells plus ECM) mechanics modeling. Quantifying the biomechanical response of soft tissue material such as the liver is complicated because of its viscoelastic, non-linear, and hysteretic behavior. 

The most basic poroelastic model of Biot [113] provides the simplest representation, which is approximately valid for small deformations. The previous focus of biomechanics has been on other tissue types, such as cartilage, intervertebral disks, and cortical versus trabecular bone. Generalizations of Biot’s biphasic model with non-linear responses have been extensively and systematically applied to cartilage mechanics modeling by Mow, Mak, and coworkers, starting with their seminal papers, Mow et al. [114] and Mak et al. [115]. In these works, a wide variety of confined and unconfined viscoelastic stress relaxation and creep experiments on cartilage have been analyzed, providing a comprehensive basis for viewing liver biomechanics responses. Suh and Spilker [116] further considered nonlinear phenomena under finite deformation. At a smaller scale, Guilak and Mow [117] applied Biot-like theory to the near-individual cell mechanical environment. These analyses of cartilage viscoelastic behavior provide excellent insight into aspects of liver viscoelasticity via parameter rescaling.

To address the mechanical responses of soft tissue, studies on the adjustable properties of polymer-gel networks and hydrogels represent an ideal starting point. As mentioned by Jain et al. [12], the use of controllable matrix properties via polymers and hydrogels allows for a systematic tissue engineering approach for investigating the role of matrix effects. A paper by Strange et al. [118] clarifies competing viscoelastic contributions to deformation of hydrogels, while Xu et al. [119] studied the biaxial stress relaxation of collagen gels. Noailly et al. [120] presented a poroviscoelastic description of fibrin gels, while Gutierrez and Groisman [121] studied tunable-strength silicone gel in microfluidic devices via atomic force microscopy. Van Oosten et al. [122] compared two different semi-flexible biopolymer networks to uncouple shear and uniaxial moduli, observing compression-softening and stretch-stiffening behaviors.

### 3.2. Liver Biomechanical Characteristics

Numerous studies on 3D tissue responses to applied stress have been developed for liver soft tissue behavior. 

Mechanical (deformation) testing of liver samples involves adding a static or oscillating compression load to an unconfined or partially confined sample. If the physical size of the load is much smaller than the size of the sample, then this is termed indentation loading. Typical sizes of in-vitro liver samples are a few mm to cm sizes, e.g., as used in Gao et al. [123]—thus containing numerous lobules. Alternatively, indentation loading can be applied to a full liver sample at various locations. For example, Jordan et al. [124] studied such loading in conjunction with imaged-based ultrasound tissue displacement. Additionally, tension tests (i.e., extension) validated with micro-CT were conducted by Shi et al. [125]. Kemper et al. [126] also explored aspects of liver tensile loading to failure, emphasizing rate-dependent effects.

Numerous studies on the biomechanical response of liver tissue have been reported in the literature. When comparing such results, one should be aware of the sample size, loading protocol, and deformation detection system employed. The liver tissue response is rate-dependent and dependent on stress history, such that measurement conditions should be clearly reported for each comparative experimental result. Levental et al. [127] constructed a simple indentation device for measuring the micrometer-scale stiffness of soft tissue, which combines two commercially available technologies. Evans et al. [128] developed a nano-indentation device useful for localized small strain testing. As the liver’s loading response can be complex, different experiments probe different aspects of the liver structure. Liver tissue has been found to be slightly non-isotropic [129]. Kemper et al. [130] showed that liver properties are strain rate-dependent and that failure stress increased with the loading rate, while failure strain decreased with the loading rate. Chen et al. [131] showed the microstructural changes occurring with increased loading and failure. Cai et al. [132] explored the creep behavior of the liver under indentations with a laparoscopic grasper used in minimally-invasive surgery. 

Several investigations have attempted to decouple the individual role of cells and ECM to mechanical stiffness by the decellularization of liver tissue. This has been accomplished by high shear stress [133] or chemical means [134,135]. This technique, which preserves the ECM structure, allows for the testing of both the acellular flow and mechanical properties of liver tissue [135,136].

Kerdok et al. [111] investigated the role of perfusion on the mechanical deformation response of the liver at the full organ scale. Here, perfused conditions resulted in increased deformability. It would be useful to pursue these studies at smaller (tissue-level) scales investigating the effects of flow through capillaries on deformation.

### 3.3. Disease Effects on Liver Biomechanics 

Here, we summarize the liver’s basic inflammation response leading to fibrosis and chronic liver injury [137,138,139]. Inflammation of the liver begins with an antagonizing agent attack on hepatocytes. This antagonizing agent can be naturally occurring (NFALD, HBC, and HCV) or artificially injected (EtOH, TAACCl4, and APAP). The latter substances are often used for research characterization of the inflammation response. 

The “injured” hepatocytes thereafter chemically signal Kupffer cells (also called macrophages, representing 15% of lobule cell types) to begin the inflammation response. These cells produce both pro-inflammatory (e.g., TNFa) and anti-inflammatory (e.g., TGFb) soluble-diffusing chemicals. As the production of TGFb tends to inhibit the production of further TNFa, typically, there is a rapid spike in TNFa production, followed by a significant decline, as TGFb increases and stabilizes. This is characterized by an extended period of TGFb availability. 

Hepatic stellate cells (HSC—5% of lobule cell types) are converted to myo-fibroblasts and portal fibroblasts in the presence of pro-inflammatory TNFa, and these cells in turn generate collagen when stimulated by the anti-inflammatory TGFb agent. Portal fibroblasts are naturally located near the portal inlet zones and are responsible for high fibrotic responses (e.g., portal hyper-tension), while myo-fibroblasts are more randomly distributed throughout the lobule. Net collagen levels are further controlled by protein-regulating matrix-metalloproteins (MMP). Collagen deposition typically occurs over a much longer time frame than the initial pro-inflammatory response. We note that increased fibrosis can limit the O_2_ distribution in the lobule, and as O_2_ is naturally lower near the central outlet, we can expect increased hypoxia and cell necrosis here with fibrosis.

An atomic force microscopy (AFM) micro-indentor was used to measure the increase of individual hepatocyte cell stiffness in cirrhosis [140]. Gaudio et al. [141] explored how cirrhosis affects liver lobule zonation, while Johnson et al. [142] quantified how cirrhosis affects drug clearance.

Steatotic livers have significantly increased fat storage vesicles (intracellular trigycerides) within hepatocytes. This increased cellular hepatocyte volume leads to possible changes in tissue mechanical properties, although the magnitude of the observed effect is, to date, controversial. Chalasani et al. [143] quantified steatosis histologically, while Nativ et al. [144,145] developed a useful defatting protocol that could prove useful as an additional source of donated livers. Yarmush et al. [146] used computational analysis to study the intracellular clearance of these trigycerides. From an analysis of biopsies from very obese subjects, Wattacheril et al. [147] determined that phospholipid zonation is reduced as steatosis converts to steatohepatitis (NASH). Interesting images of resulting lobular zonation patterns are shown in this work. 

The liver’s basic inflammation response can also represent a first step in cancer development through the activation of pro-inflammatory signaling [148,149,150] Conversely, the alteration of this signaling pathway shows promise in reversing fibrosis and Hepatocellular carcinoma (HCC) [151].

Cancer cell stiffness has been probed (and differentiated from normal cells) using AFM [152], while the contribution of changes in ECM to cancer stiffness has been studied using particle tracking microrheology [153,154]. Netti et al. [155] described the role of ECM mechanics in determining interstitial transport in solid tumors. A non-equilibrium thermodynamic model for tumor ECM, including degradation, has been developed by Xue et al. [156]. Hoyt et al. [157] quantified tissue elastic parameters, while Xue et al. [158] developed a comprehensive model for the coupled biochemical-mechanical poroelasticity of tumors.

Butler et al. [159] described how biomechanics impacts tissue regeneration. The link between liver fibrosis and hepatocellular cancer has been emphasized by Philips et al. [151] and Rahbari et al. [160].

### 3.4. Summary

In contrast to flow bioreactor systems, mechanical liver bioreactor studies typically employ either artificial hydrogel matrices or liver tissue slices. Both are characterized by ill-defined tissue structures and the biphasic numerical modeling of such systems most often utilizes a porous-media-like representation of the mechanical-flow process, whereby the sinusoidal structure is averaged. In later portions of this report, we shall introduce a more exact dual continuum numerical approach, which can also be applied to mechanical modeling.

## 4. Lobule Models: Zonation, Tortuosity, Fibrosis, and Mechanics as Viewed through Computational Methods

### 4.1. Computer Lobule Models

Tissue engineering constructs are generally devised to mimic in-vivo conditions as much as possible. Computational models provide a further perspective on the desired in-vivo state and the effects of possible parameter variations. Here, we focus on the liver lobule as the “gold standard” for tissue engineering constructs to mimic.

Numerous research groups have explored aspects of the liver lobule with computational methods. Both pseudo-steady state and kinetic models of cellular metabolism have been employed, with variable numbers of cellular metabolites and rate processes being considered in these models. Since hepatocytes vary in enzyme expression and function, depending on their spatial location in the lobule (zonation), many authors have extended their metabolic treatments to pseudo-1D flow descriptions along a representative sinusoid. When considering zonation, typically 8 to 10 compartments along the sinusoid are considered. Complications such as blood non-Newtonian viscosity and the Fahraeus–Lindqvist effect are normally not considered in these flow models, although feasible [161].

Chalhoub et al. [162,163] used both steady state and kinetic representations of carbohydrate and fatty acid metabolism with approximately 20 metabolites to analyze a 1D distribute model along a liver sinusoid at rest and during exercise. Calvetti and coworkers [164,165] later extended their steady state analysis utilizing Bayesian flux balance methods to estimate parameters and extrapolate to kinetic models.

König et al. [166] and Bulik et al. [167] utilized an extensive kinetic model with approximately 50 metabolites to analyze the roles of hormones (insulin, glucagon, and epinephrine) and allosteric effects on glucose homeostasis. They later extended this approach to consider zonation via rescaling protein abundances (and thus effective reaction rates) along the periportal-pericentral axis [168]. This allowed an analysis of glycogen variation during a fasting-refeeding schedule. In follow-up work [169], they developed a kinetic model for protein turnover and applied this to various feeding and hormone states.

Ohno et al. [170] presented a detailed analysis of the urea cycle and how the zonal expression of three critical enzymes affects the production of urea and glutamine along eight compartments on a 1D flow path between the periportal and pericentral lobule locations. Thereafter, Schliess et al. [171] developed a simple five-component model of 1D zonated ammonia detoxification of the liver during damage and regeneration and extended this to an array of seven lobules. This model was subsequently applied to devise a therapeutic strategy to reduce hyperammonemia by the injection of a missing glutamate dehydrogenase (GDH) enzyme (see Ghallab et al. [172]).

Steatotic livers have significantly increased fat storage vesicles (intracellular trigycerides) within hepatocytes. This increased cellular hepatocyte volume leads to narrowed and tortuous microvessels and reduced sinusoidal blood flow and O_2_ availability, and eventual hepatocellular necrosis [173]. This simple (unzoned) model was later extended to consider zonation aspects of fatty acid uptake, by Schleicher et al. [174,175]. These kinetic models utilized approximately five metabolites and a similar number of kinetic reactions.

Hijmans et al. [176] conducted a review of the consequences of combined glucose and fatty acid zonation for insulin resistance, steatosis, and non-alcoholic fatty liver disease. These observations were quantified more completely in work by Ashworth et al. [177,178], who developed a 1D zoned eight-compartment model of the liver lobule with detailed kinetics. This extensive model considered approximately 20 components and reactions, as well as protocols for adjusting feeding schedules. A useful quantitative analysis of metabolic changes in steatosis and NAFLD followed, emphasizing, for example, the role of increased SREBP-1c expression in this metabolic disease. Finally, Noorman et al. [179] extended the Ashford model to assess the effects of dispersion and the number of compartments.

In summary, although allowing a limited treatment of zonation, these 1D models cannot predict blood velocity variation and complex flow patterns occurring throughout a lobule. 

Other researchers have investigated 2D and 3D flow models to address the consequences of liver lobule flow patterns and zonation. Debbaut et al. [180] utilized CFD methods to calculate flows through an imaged 3D sinusoidal network with a size of 50 µm^3^ to study the effects of an anisotropic sinusoid network pattern. Ding et al. [181] utilized a representation of the liver acinus with an explicit sinusoid pattern to analyze the blocking effects of cancer cells on a liver-zoned O_2_ distribution. Hoehme et al. [182] emphasized the role of sinusoid microvessels in determining lobule architecture for liver regeneration.

The majority of 2D and 3D computer lobule models use a porous media approach, which is an upscaling concept without explicit representation of the sinusoid flow paths. Rather, a representative volume is assumed to consist of an average of tissue and sinusoid volumes. Using this approach, Bonfiglio et al. [183] studied the factors affecting the flow patterns across an array of 2D hexagonal lobules—factors such as anisotropy, blood shear thinning, and mechanical deformation. Later, they extended this approach to investigate coupled blood flow and lymph production [184]. Debbaut et al. [185] considered an isolated 3D porous media liver lobule and investigated the importance of anisotropic flow and vascular septa on the predicted perfusion. Ricken et al. [186,187] developed a 2D biphasic porous media model (i.e., including deformation) to investigate the zonation of glucose, lactate, and glycogen metabolism of the liver lobule during cycles of feeding/fasting. Hu et al. [188] studied the predicted effects of fibrosis and cirrhosis on 3D flow patterns using a porous media model.

In summarizing this section, it should be emphasized that computational lobule models need to be generalized to an array of lobules to be representative of experimental tissue plugs often used to probe the liver function. This requires one level of computational upscaling for efficient calculations. Further comments emphasizing this point of view are presented below.

Lerapetritou et al. [189] presented an overview of the modeling of xenobiotic metabolism in the liver which includes various approaches to approximating sinusoid structure and mixing effects. Yan et al. [190] proposed an agent-based technique to investigate the role of lobule microarchitecture feature sinusoid structures on predicted drug metabolism and production characteristics. Thereafter, Wambaugh and Shah [191] similarly considered variations of explicit idealized sinusoid 2D distributions within a hexagonal lobule and their impact on the production of non-metabolized and metabolized chemicals, also using an agent-based model. 

Sluka et al. [192] developed a multi-scale approach to modeling xenobiotics, utilizing a 1D model for the lobule (pipe model). They applied their model to acetaminophen metabolism. Later, the same group, Fu et al. [193], extended their lobule model to a 3D model with explicit sinusoid (“NET”) representation. Cherkaoui-Rbati et al. [194] presented a full-body physiologically based pharmacokinetic (PBPK) model for drug–drug interactions which incorporated a 2D hexagonal model of the liver lobule with an explicit sinusoid pattern. They used this model to examine the drug–drug interaction of 10 drugs with co-injected midazolam, which is a common drug relaxant. 

### 4.2. Tortuosity, Percolation, and Tumour Transport Issues

Tortuosity (dimensionless) is a measure of the twisted nature of an arbitrary curve, i.e., the ratio of the arc length of a curve relative to its end-to-end distance. Here, we apply it to characterize the nature of fluid flow paths around objects and through conducting channels. Tortuosity is a fundamental characteristic of liver tissue and sinusoids that is typically not captured by bioreactor design. To our knowledge, to date, microfluidics and even bioprinting have not been able to realistically capture this aspect of tissue engineering representation. Changes in tortuosity are also a dominant hallmark of serious liver disease, such as cirrhosis and cancer. 

Only truly 3D representations of tissue architecture allow such analysis, and computational models beyond CFD methods are best able to explore these factors. Early creative work by Issa [195] explored the role of tortuosity and percolation experimentally, but the manufactured percolation reactors need to be miniaturized further for more direct applications to quantitative liver flow results. Calculational methods based on Darcy flow appear to be best able to capture tortuosity effects and their changes in a straightforward, practical way.

Tortuosity effects are multi-scale. In the space-of-Disse surrounding individual hepatocytes, flow paths around random collagen fibers determine the permeability [196,197]. This leads to a dramatic porosity dependence of permeability, especially at a high porosity. Alternate permeability representations, based on the Carman–Kozeny equation originally developed for flow around sand grains, have also proven useful [198,199], but with a stronger dependence of tortuosity on porosity. Pedersen et al. [200] used this to investigate the role of ECM fiber architecture on the resultant fluid shear stress experienced by cells generated from fluid flow.

Tortuosity increases dramatically as porosity decreases and flow path blockage increases (approaches the percolation limit) [201,202,203]. Here, these models are first developed for relatively isotropic blocking materials (e.g., sand grains), but similar concepts hold for extended structures such as fibers [204].

An alternate view of flow models (perhaps more appropriate at the scale of the lobule) is to consider the density and connectivity of vessels. Here, ideas of fracture network models allow an analysis of flow from percolation limits (non-connectivity of vessels) to highly vascularized connected situations [205,206,207]. Similar concepts have been employed in describing brain vasculature networks [208].

Changes in the vascular network structure (tortuosity) between normal and tumor tissue have been imaged and analyzed in early work by Jain and coworkers [209,210,211,212]. They emphasized that normal tissue micro-vasculature is “space filling”, while tumor vasculature is “fractal”. Comparing the 2D fractal dimension of images to models which generate equivalent fractal dimensions (space filling growth for normal tissue and invasion percolation for tumor tissue) allowed these differences to be quantified. Craciunescu et al. [213] confirmed these results using contrast-enhanced MRI. Herman et al. [214] proposed how vascular tortuosity changes with tumor growth. Netti et al. [215,216] developed a model for interstitial pressure and fluid flow in tumors.

### 4.3. Biomechanics Computer Models

Even for soft tissues such as the liver, it is expected that basic Biot-type poroelastic behavior is recovered in the limit of small deformations. Linear elastic models utilize two coefficients, e.g., Young’s modulus E and Poisson’s ratio ν, to describe elastic deformation behavior. Young’s modulus is a measure of elastic stiffness, while Poisson’s ratio describes the degree of lateral expansion with axial compression (the limit ν = 0.0 represents completely compressible material, while ν = 0.5 represents incompressible material, such that their volume is essentially constant).

Representative liver values are E = 5 kPa and ν = 0.35 [217]. Extensive fibrosis will increase liver E values significantly, perhaps by five times [218]. This compares to cartilage values E = 10,000 kPa and ν = 0.167, while water has an even larger Young’s modulus E = 2,300,000 kPa and ν = 0.5 [219]. Note that other elastic moduli (bulk modulus, shear modulus, aggregate modulus, Lame’s constants, etc.) can all be expressed in terms of E and ν. In particular, the shear modulus G = E/(2(1 + ν)).

For fluid-filled (porous) media, Biot’s theory requires two additional constants describing fluid-solid coupling: Biot’s constant α and Skempton’s coefficient Sk (see Rice and Cleary [220]). These can also be expressed in terms of other coefficients (e.g., drained Poisson’s ratio). For the liver, we chose α = 1.0 and Sk = 1.0. Finally, the relaxation of fluid stress with fluid flow is governed by a “pressure diffusion coefficient”, which is essentially a rescaled Darcy permeability. We will make further comments on the role of this coefficient in subsequent sections. 

Recognizing the inherent highly deformable viscoelastic properties of soft tissues, there are two fundamental approaches for extending this basic constitutive model: a phenomenological approach with fitting directly from experimental data, and a physics-based approach where strain is calculated from accepted physical laws. Marchesseau et al. [221] reviewed many such models.

The simplest (and most popular) phenomenological model is a Maxwell model with elastic spring and viscous dashpot contributions in parallel. Kerdok et al. [111] have used such a model to model small strain frequency indentation tests and large strain creep indentation tests. Basdogan [222] reviewed her work on fitting this model to the changes of mechanical behavior of human and animal livers with a degree of fibrosis. Note that a Maxwell model with one relaxation time constant is essentially equivalent to Biot’s theory.

Physics-based constitutive models of the liver attempt to represent the hyperelastic contribution of collagen fibers, the viscoelastic behavior of proteoglycan (GAG), and the Darcy-like flow behavior of interstitial fluid. Hyperelastic models have their origin in the large stretch behavior of rubber elastic fibers [223]. Vande Geest et al. [224] developed a porohyperelastic model for general soft tissues. Sparks and Dupaix [225] proposed a rate-dependent large deformation model of the liver response to blunt impact loading, also based in part on polymer mechanics concepts. Moran et al. [226] numerically modeled aspects of liver poro-hyperviscoelastic behavior. Perepelyuk et al. [227] demonstrated the changes fibrosis induces to liver biomechanics and developed a model for the shear strain softening and compression stiffening of fibrotic livers. Untaroiu [228] considered the liver injury response to tensile loading via rate-dependent hyperelastic (Ogden) material parameters matched to experiments. Xue et al. [229] considered GAG swelling effects in a nonlinear poroelastic theory of normal tissues and tumors.

### 4.4. Basic Concepts Illustrated with Ideal Models

Mathematical modeling allows experiments to be put into a consistent theoretical framework. Indeed, it is a fruitful way to combine points of view from the multi-disciplinary approach used in tissue engineering. 

**Figure 1:**
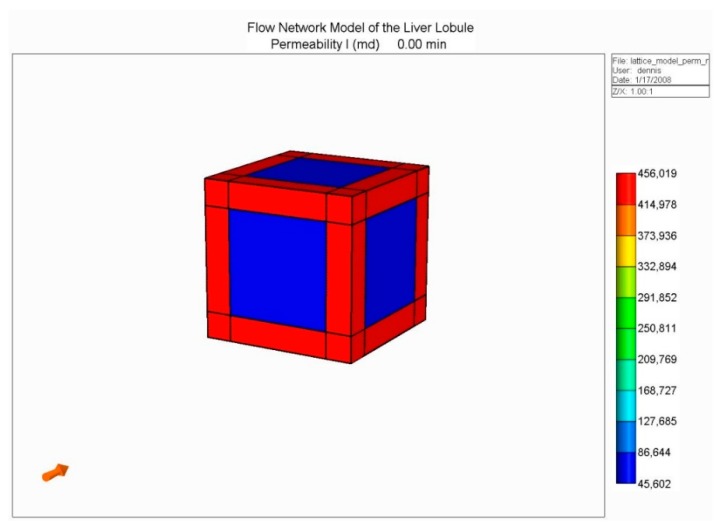
Base case flow element: Sinusoid local network (with flow and mechanical properties) surrounding cell + extracellular matrices (ECM) cube (with different flow and mechanical properties).

To summarize many of the concepts outlined in this review, we now present some simple simulations of fluid flow and mechanics at the lobule scale, based on our earlier work [230,231,232].

(A): Figure 1 shows our basic conceptual unit. This consists of a single-cell unit surrounded by ECM, and a local portion of an idealized sinusoid network surrounding this. Volumes are calculated using a 3 µm grid size for the sinusoid and 24 µm grid size for the tissue. Basic physics concepts are used to estimate physical properties for this unit. The sinusoid porosity and permeability are estimated via a circular tube of radius fitted inside a square cross section of the side dimension 2R (Poiseuille’s law [233]). The tissue permeability uses a Carmen–Kozeny correlation as a function of porosity. This was originally developed for packed beads [234], but has been found to also be appropriate for soft fibrous media [199]. Here, the fibrous media is assumed to have formed by mixtures of collagen fibers (approximate size of a 50 nm diameter and 300 nm length). It is noted that diffusion in a sinusoid is assumed to be free diffusion (based on the particle size and the Stokes–Einstein equation), while diffusion in tissue grid cells is less free, due to restricted motion around collagen fibers [235,236,237]. Metabolic reactions are assumed to follow Michaelis–Menten formulation [238].

**Figure 2:**
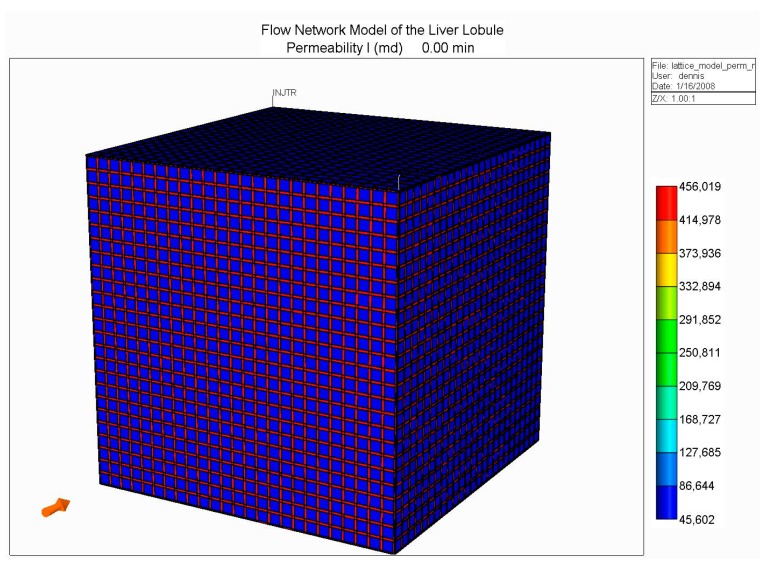
Idealized 3D flow network structure: Lobule lattice.

Basic flow and mechanical properties of this unit are summarized in Table 1.

(B): Next, we constructed an idealized lobule by combining 50 × 50 × 50 units from our basic element, the result of which is shown in Figure 2. Such an approach was first outlined in our earlier papers [230,231], but for ¼ element in 2D. Here, there are four inlet (portal) ports and one per-central outlet port, as illustrated in Figure 2.

With this model, we developed models for O_2_ utilization and paclitaxel drug metabolism, utilizing Michaelis–Menten kinetics, as presented in our earlier work, but here focusing on the effects of fibrosis on predicted metabolic behavior. As indicated earlier, fibrosis affects both the hydraulic permeability of the tissue surrounding individual hepatocytes (micro-tortuosity—the tissue permeability value in the flow element of Figure 1), and the sinusoid network connectivity (structural tortuosity) throughout the liver lobule (i.e., the hydraulic sinusoid permeability of the network of Figure 2). Our earlier work has demonstrated that the latter effect dominates the overall flow of the lobule.

To illustrate this more quantitatively, four cases are compared: (i) an idealized, fully-connected sinusoid network with a constant sinusoid hydraulic permeability; (ii) a sinusoid network with a reduced percolation fraction of 0.7; (iii) a sinusoid network with a reduced percolation fraction of 0.4; and (iv) a sinusoid network with a reduced percolation fraction of 0.2. These latter cases represent increased levels of fibrosis. Although not shown, we have conducted additional simulations with random variations in individual sinusoid element permeabilities around the employed mean value, as well as reduced tissue hydraulic permeabilities, but the percolation effect dominates the observed flow patterns. Our earlier two papers discuss this in greater detail (but for 2D models). All simulations were conducted at a fixed pressure drop between the periportal and pericentral inlet/outlet.

**Figure 3:**
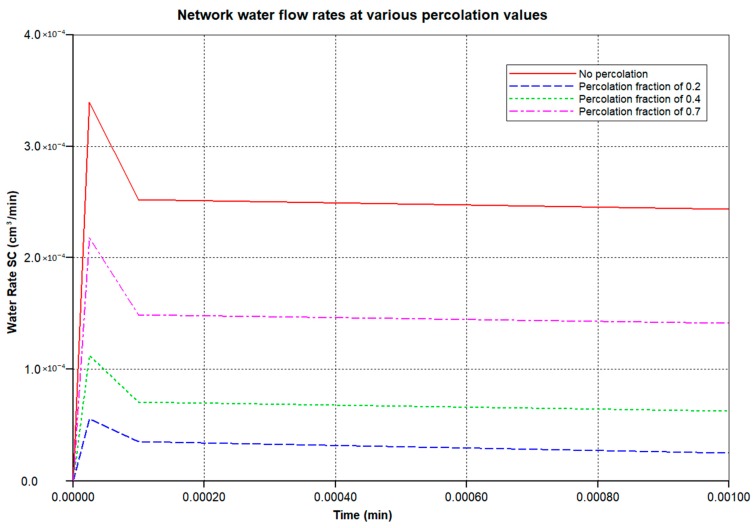
Network water flow rates at various percolation values. O_2_ production: after 3 min.

**Figure 4:**
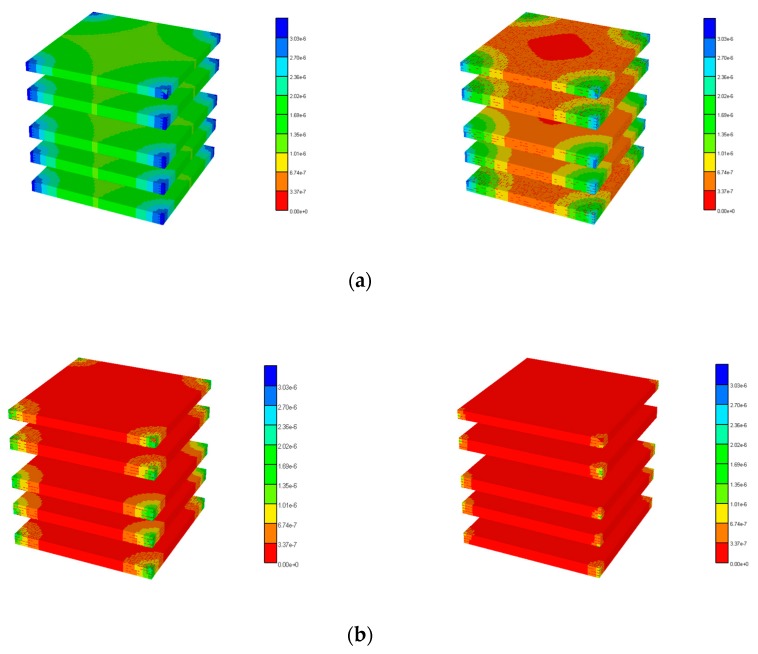
3D O_2_ distribution levels at various percolation values. (**a**) Basic and high percolation. (**b**) Mid and low percolation.

Figure 3 illustrates the reduced flow reduction with increased fibrosis at a selected periportal inlet (this is essentially identical at all inlet ports). The reduced flow results in delayed and reduced pericentral O_2_ production with fibrosis. An overall increase in O_2_ utilization is found as metabolism then dominates convection. Figure 4 shows selected slices of the steady state O_2_ distribution in the 3D lobule for these cases.

As the resulting oxygen distribution is believed to affect the zonal expression of CYP enzymes, we utilized the steady state O_2_ distributions to estimate fibrosis modifications of drug-metabolizing (paclitaxel) enzymes across the lobule. Here, we followed the protocol outlined in our earlier paper [232] to calculate these zonal distributions.

**Figure 5:**
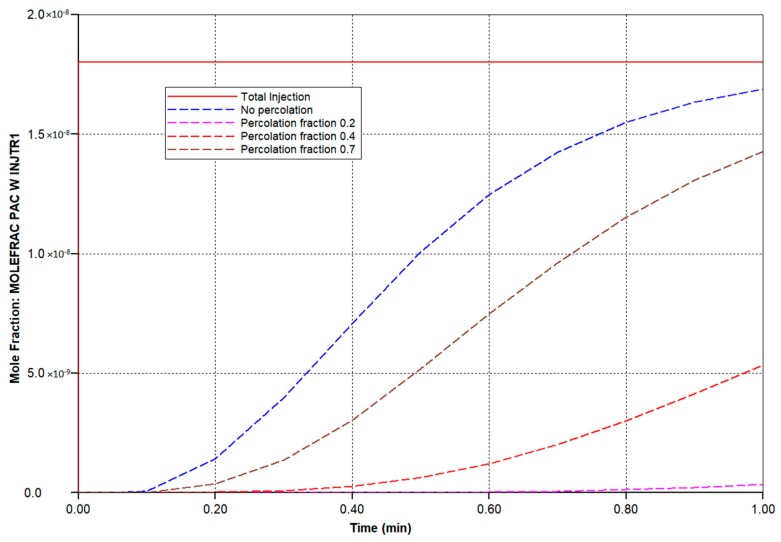
Injected/produced PAC and PAC-OH at various percolation values.

With these predicted levels of paclitaxol (PAC) metabolizing enzymes, a first-pass injection of paclitaxel was investigated under increasing fibrosis. Figure 5 compares injected PAC and produced levels of PAC and hydroxypaclitaxel (PAC-OH) over time at various percolation values. For the basic case, no PAC is produced and metabolized PAC-OH is still approaching injected PAC levels after 1 min. For the 0.7 percolation case, produced PAC-OH is below that of the basic case due to the lower flow rate with some fibrosis. As fibrosis increases further (0.4 and 0.2 percolation values), the flow rate decreases more significantly and less and less PAC-OH is produced at 1 min.

**Figure 6:**
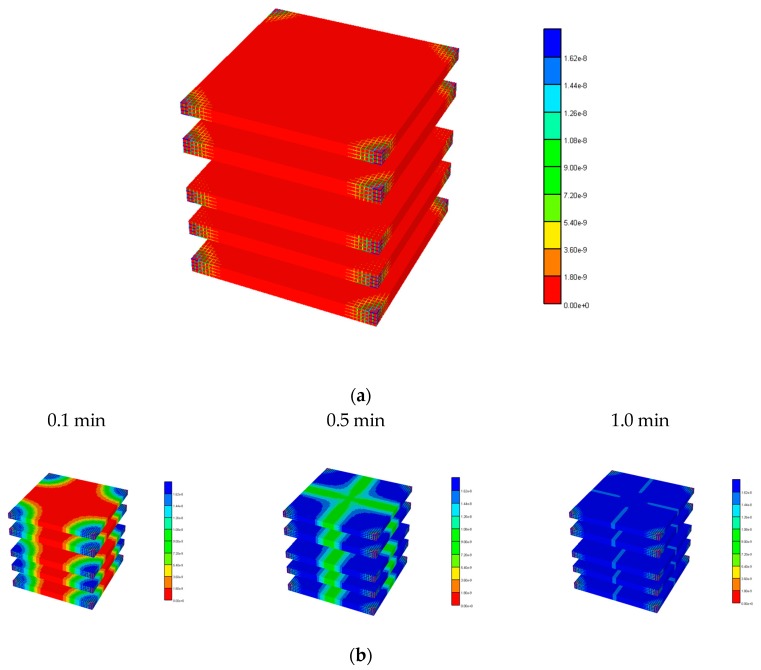
3D PAC and PAC-OH distribution levels for the basic lobule model. (**a**) PAC distribution for the basic lobule model at 1 min. (**b**) PAC-OH distribution for the basic lobule model at various times.

**Figure 7:**
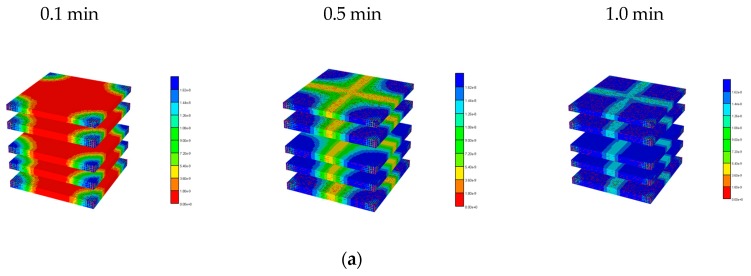

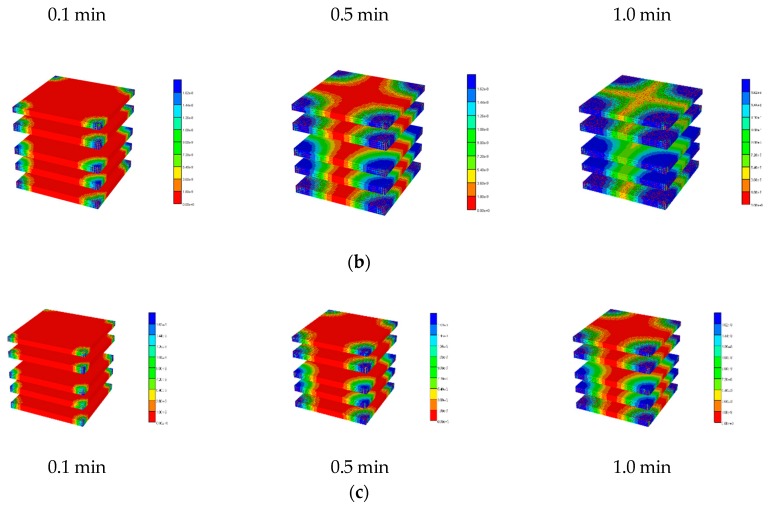
PAC-OH distribution levels at various percolation values. (**a**) PAC-OH distribution for the 0.7 percolation value at various times. (**b**) PAC-OH distribution for the 0.4 percolation value at various times. (**c**) PAC-OH distribution for the 0.2 percolation value at various times.

Figure 6 illustrates the spatial distribution of PAC at 1 min and the evolution of PAC-OH spatially over time. Figure 7 shows the equivalent PAC-OH distributions for various percolation levels. The equivalent PAC distributions for these cases are not shown explicitly, but are limited very near the injector ports at levels that become lower as percolation increases (i.e., lower than that shown for the basic case in Figure 6a).

(C): To dynamically simulate the mechanical response of tissues, we will couple a fluid flow simulation with a continuum finite element simulator. This uses an efficient numerical algorithm termed “iterative coupling” [240], such that the two independent simulation codes generate sequential fluid flow and mechanical responses that impact the other process at a given time step, both of which are iteratively converged before proceeding forward in time. This algorithm has been applied to numerous geomechanical problems associated with subsurface oil production, but has also been utilized to describe the mechanical responses of bone and the intravertabral disk to various mechanical loading scenarios [241,242,243,244,245].

Here, we analyze the stress relaxation of our idealized liver lobule model, as a predicted response of an applied nano-indentor. Again, we will focus on the impact of fibrosis on the lobule mechanical response. Such samples exhibit biphasic creep and stress relaxation responses due to coupled elastic solid and fluid flow phenomena. As such, the competition between stiffness (Young’s modulus) and flow (hydraulic permeability) is paramount. Biot’s poroelasticity requires four mechanical parameters: two solid elastic parameters (Young’s modulus and Poisson’s ratio) and two fluid-solid coupling parameters (Biot’s constant and Skempton’s coefficient). Extensions can include non-linear elasticity, e.g., Young’s modulus. 

**Figure 8:**
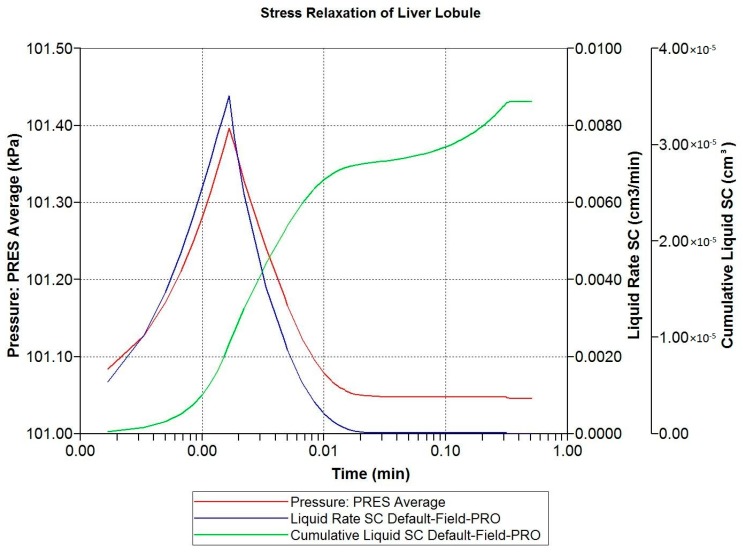
Stress relaxation responses: Average pressure and fluid expulsion (logarithmic scale).

**Figure 9:**
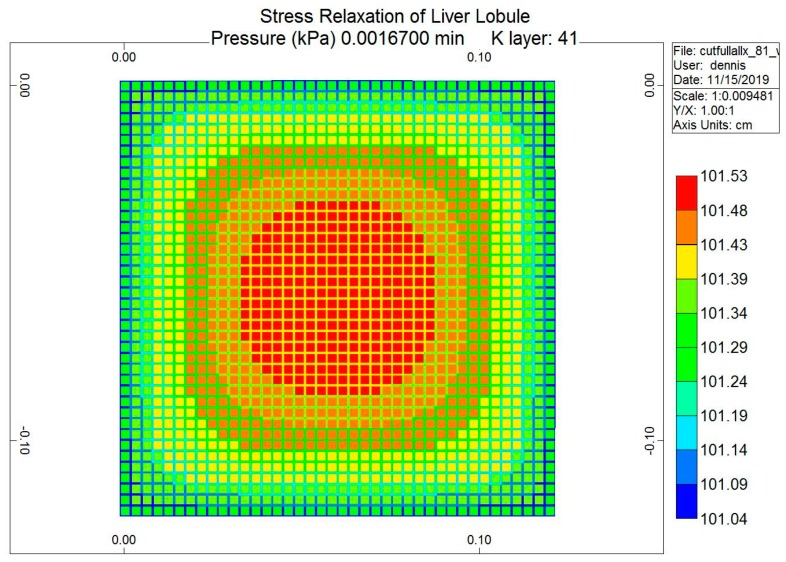
Stress relaxation responses: Average pressure at maximum displacement (2D).

**Figure 10:**
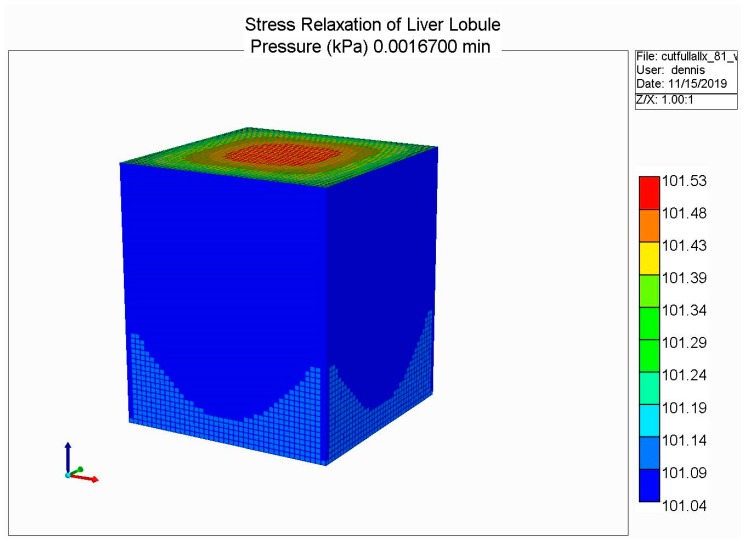
Stress relaxation responses: Average pressure at maximum displacement (3D).

**Figure 11:**
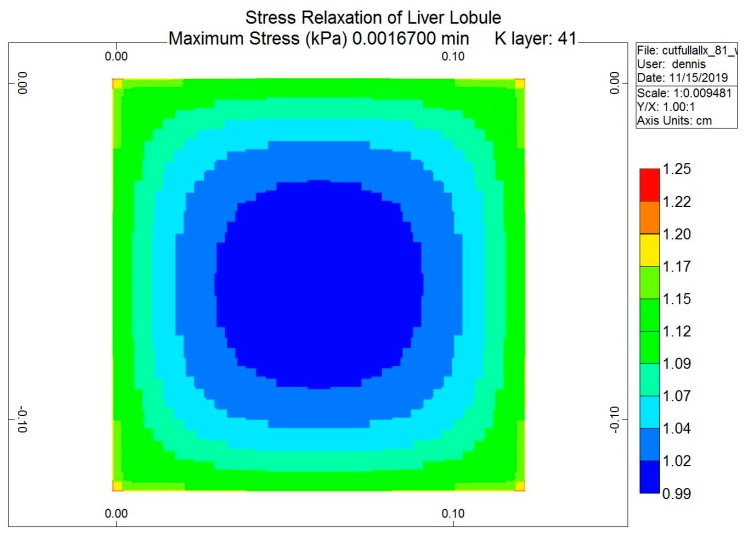
Stress relaxation responses: Maximum stress (Z) at maximum displacement (2D).

**Figure 12:**
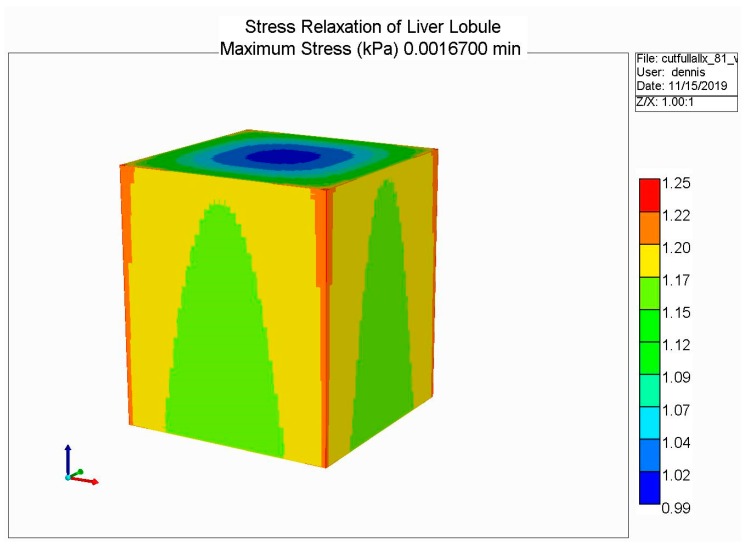
Stress relaxation responses: Maximum stress (Z) at maximum displacement (3D).

The loading protocol is to apply a 5% displacement of the top of the lobule for 0.1 s (at a rate of 0.5 s^−1^), followed by stress relaxation over the subsequent 1 min. Figure 8 shows the predicted fluid pressure responses using a logarithmic time axis, including the net fluid outflow from the sides of the lobule, which provides the stress relaxation. More mechanistic details are shown in selected spatial plots at the time point of maximum applied displacement (0.00167 min). Figure 9 and Figure 10 show, respectively, the pressure distributions at a selected depth and in full 3D. Here, the distinct pressure responses in the sinusoids and tissue are observed. Additionally, the release of pressure at the boundaries is noted, due to induced fluid outflow. Numerous measures of stress distributions within the lobule can also be visualized. Here, we show the maximum (Z) stress distribution in 2D and 3D in Figure 11 and Figure 12, respectively. Although the material is isotropic and the applied displacement is uni-directional, fluid outflow at the boundaries creates a 3D response. For this case, the magnitudes of predicted pressure and stress responses are small because of the small size of the lobule element. Upscaling to a tissue-plug size (a collection of numerous lobules) would give larger observable effects. 

**Figure 13:**
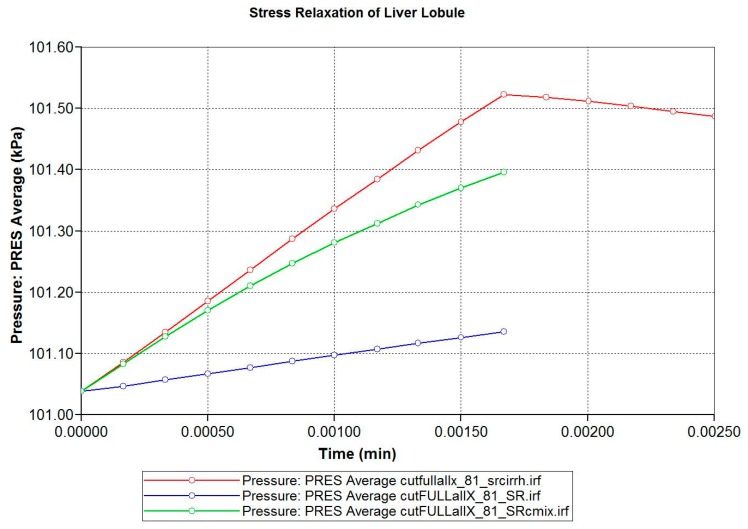
Comparative stress relaxation responses: Average pressure during loading (linear scale).

Finally, Figure 13 compares the loading response period for three cases: (i) normal lobule response; (ii) fibrotic lobule response with increased stiffness and reduced hydraulic permeability; and (iii) a mixed case with increased stiffness, but normal permeability. This latter case allows a comparison of mechanisms. These simulations taken together indicate that fibrosis-induced increased stiffness results in an increased stress rise, while fibrosis-induced decreased permeability also contributes by inhibiting fluid outflow.

## 5. Conclusions

### 5.1. Practical Applications

There are numerous practical applications of these studies and concepts that tissue engineering has contributed to and will continue to advance. The development of bio-artificial livers (BAL) for maintaining patient health in the absence of a liver transplant illustrates a practical application of liver zonation. Catapano and Gerlach [246] and Gerlach et al. [247] provided an overview of the issues associated with the development of such devices, as well as a comparison of available BAL devices, as of 2008. These articles also mention the seminal pioneering work by the research groups of Sussman, Morsiani, Miller, Dimitriou, and Bader in this field.

Most such devices are based on a hollow fiber design and utilize rat or porcine hepatocytes as widely available cell lines. Issues include oxygen and nutrient availability while ensuring minimal shear stress on hepatocyte cells, normally addressed through the use of a protective permeable membrane. Sauer et al. [248], for example, examined the effects of temperature and mechanical stress on the viability of cells used in such bioreactors. Mathematical modeling can also be utilized to improve the basic design efficiency of such devices [249,250].

Commercial devices often use mats of fabric surrounding the hollow fabrics. This includes the MELS CEllModule system, proposed by Sauer et al. [251,252], based on the construct first developed by Gerlach and coworkers [253]. Hoffmann et al. [254] developed a miniaturized bioreactor version of this construct for further investigation. An alternate design also employing spiral wound non-woven fabric, AMC BAL, was developed at the University of Amsterdam, by Flendrig et al. [255]. Further design enhancements (e.g., Poyck et al. [256]) are often based on numerical simulations [257]. A detailed comparison of the performance of these two commercial systems was provided by Poyck et al. [258]. They concluded that the major operational factor was not the bioreactor design, but rather the choice of applied cell type.

For the liver, non-invasive clinical testing of normal and diseased liver at the scale of the full liver can be conducted via variations of ultrasound methods. These are based on the measurement of a sound wave velocity related to a tissue modulus. In particular, a shear wave velocity V_s_ (m/s) can be related to shear modulus as G_s_ = ρV_s_^2^ (Pa). Normally, a typical density ρ = 10^3^ (kg/m^3^) is assumed. Note that elastography requires a sound (vibration) source and a sound (vibration) detector. 

The most well-known method is transient elastography (FibroScan, Echosens), developed in France, which uses a 50 Hz pulse to probe a volume of approximately 4 cm^3^ of liver tissue sequentially [259,260,261,262,263]. The intent is to correlate this with the structural aspects of the diseased liver, including fibrosis, steatosis, cirrhosis, and cancer, as all conditions are expected to affect the sound velocity through liver tissue. This is then directly related to tissue stiffness, as mentioned above. 

Various related modalities include shear wave elastography [264,265], acoustic radiation force elastography [266], real-time elastography [267], and magnetic resonance elastography [268]. All of these differ in source and detector characteristics, but generally involve ultrasound (>20 kHz) frequencies. Fundamental research relating these non-invasive techniques to more traditional mechanical testing of the liver has also been investigated [269,270,271,272,273]. Konofagou et al. [274] emphasized the effects of poroelasticity on the transient elastography response by comparing observations to theoretically calculated profiles.

Acute liver failure (ALF), whatever its cause, has a high risk of mortality, and the default treatment is orthotropic liver transplantation (OLT). For example, Vilar-Gomez et al. [275] summarized the mortality risk of patients with advanced non-alcohol fatty liver disease. One main limitation of OLT is the scarcity of donor livers, including the rejection of possible candidates due to hepatic steatosis. With obesity rising in the general population, this situation is expected to worsen. New techniques from tissue engineering for liver defatting prior to transplantation provide an exciting possible solution to this issue [276,277].

Computer-aided real-time surgical training and planning used in conjunction with imaging modalities has attracted growing interest. For practical applications, accurate complex constitutive models must be combined with very fast numerical techniques [278,279].

### 5.2. Summary

Tissue engineering holds great promise for addressing the critical health issues associated with liver function and liver disease. Often, however, relevant experiments can be difficult to design, and time-consuming and costly to conduct. The use of mathematical models in all stages of process development is therefore recommended, helping with design issues, detailed matching of the experiment itself, extending the range of experimental variables studied, and finally upscaling and extrapolating the conclusions to realistic in-vivo situations.

- Basic physics laws for liver transport, reactions, and mechanics provide useful fundamental insight into all the processes of interest:
- Poiseuille’s law and Darcy flow, the Carman–Kozeny equation, Stokes–Einstein diffusion, Michaelis–Menten kinetics, and Biot poroelasticity.

Complications arising from non-linear properties and measures of variability and randomness (for property distributions). Spatial effects are dominated by vasculature network characterizations (ideal space filling versus fractals, DLA, tortuosity, and percolation).

In particular, tortuosity as a result of liver disease states provides a unifying concept in all these investigations and needs to be a focus of further tissue engineering experiments and computer modeling. Tortuosity at multiple scales, including the (i) space of Disse, (ii) lobule capillary network, and (iii) full liver vasculature, affects the performance of the liver in health and disease. Tortuosity affects or is affected by the disease states of steatosis, fibrosis, cirrhosis, and cancer and furthermore impacts the ability and feasibility of drug treatments. Computational methods based on Darcy’s flow appear to be best able to capture tortuosity effects and their changes in a straightforward, practical way.

Here, we have attempted to indicate where computer models provide insight in these areas. Further development of these computer models is also required, however, with a greater emphasis on

- upscaling (or multi-scaling) in time and space, e.g., single cell to lobule and lobule to full liver, and
- the use of dual continuum models (separate upscaled elements for tissue and sinusoids).

Most often, researchers use averaged single continuum models for upscaled fluid flow or mechanics simulation. We have highlighted some examples of this in earlier sections. Implicitly, such models are only appropriate at later timescales when mass transfer between regions has reached pseudo equilibrium.

In contrast, a computationally efficient algorithm to upscale these results to the tissue scale, and ultimately to the full organ scale, is required. This algorithm is termed dual continuum modeling, such that each averaged spatial location property is represented by one averaged tissue property value and one averaged sinusoid property value. This holds for both static properties, such as porosity and permeability, and dynamic properties, such as component concentrations. Other researchers who have attempted upscaling concepts when modeling the liver include Xie et al. [280], Schwen et al. [281,282,283], Berndt et al. [168], and Sluka et al. [192].

The algorithm in our software was originally developed to model fluid production from fine-scaled fractures in geological heterogeneous media [284], but has been utilized by other authors to represent dynamic processes occurring in the lung [285], the heart [286], or the brain [287,288].

Upscaling mechanical responses while utilizing dual or multiple continuum models has also been considered [289,290]. Within our software, a predicted mechanical response of liver tissue at the scale of several centimeters can be efficiently simulated by combining the available dual continuum and iterative coupling algorithms. Although applied to heart modeling and not the liver, work by the Segers group [291] indicates several additional upscaling techniques which should be applied to upscaling liver flows as well. Appendix A gives a brief example of dual continuum fluid flow upscaling in a full liver model based on grid cells representing either macro-vasculature or liver lobule units.

## Figures and Tables

**Table 1:** Base case flow and metabolism parameters.

Parameter	Characteristic (SI) Unit	STARS Unit
Sinusoid Porosity *Φ*_sin_	0.7854	0.7854
Sinusoid Permeability *K*_sin_	1.125 μm^2^	1.140 Darcy
Sinusoid Effective Diffusion *D*_sin_	4.2 × 10^−10^ m^2^/s	2.5 × 10^−4^ cm^2^/min
Tissue Effective Diffusion *D*_tis_	4.2 × 10^−11^ m^2^/s	2.5 × 10^−5^ cm^2^/min
Maximum Rate *v*_max_ *	0.06 μM/min	1.08 × 10^−9^ molefrac/min
Half Saturation Constant *K*_m_ *	10.0 μM	1.8 × 10^−7^ molfrac
Linear Rate *v*_max_/*K*_m_ *	6.0 × 10^−3^ min^−1^	6.0 × 10^−3^ min^−1^
Blood Viscosity	3.5 × 10^−3^ Pa s	3.5 cpoise
Young’s Modulus E	5 × 10^3^ Pa	5 kPa
Poisson’s Ratio, *v*	0.35	0.35
Biot’s Constant, α	1.0	1.0
Skempton’s Coefficient Sk	1.0	1.0
(1 Darcy = 0.9869 µm^2^ in engineering permeability units) * Paclitaxel kinetic elimination Michaelis–Menten parameters converted from Vaclavikova et al. [239], their Table 4.		

**Figure A1:**
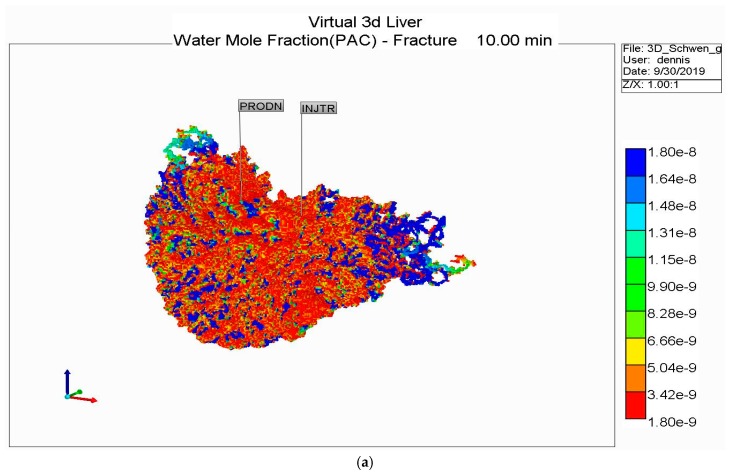

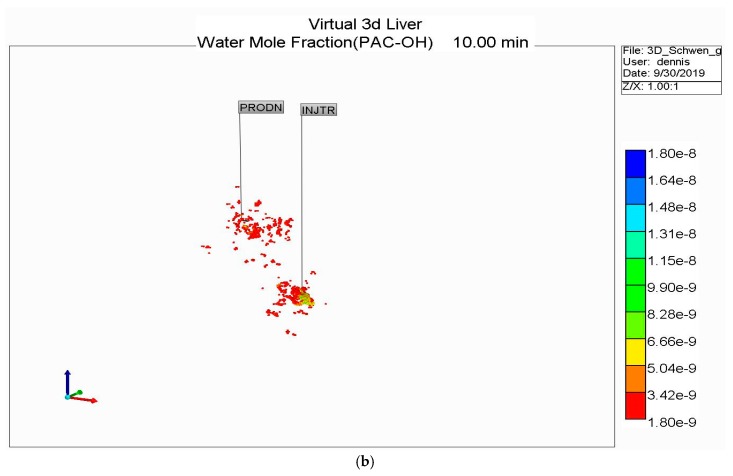
Reactive PAC and PAC-OH distributions in a dual continuum model (no diffusion). (**a**) Reactive PAC distributions in the fracture after 10 min (no diffusion). (**b**) Reactive PAC-OH distributions in the matrix after 10 min (no diffusion).

**Figure A2:**
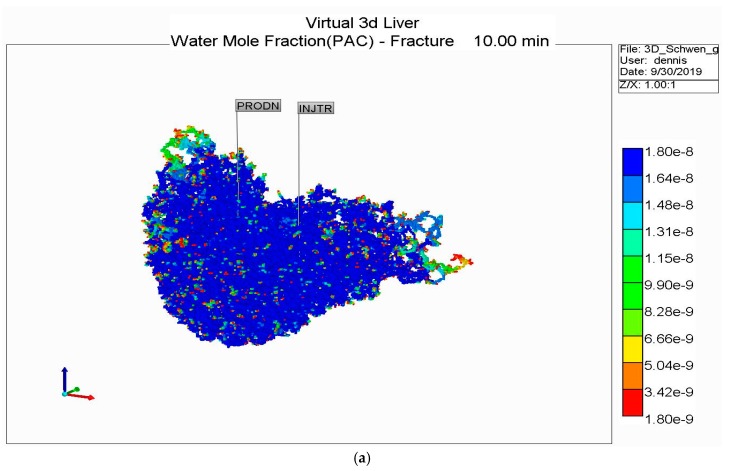

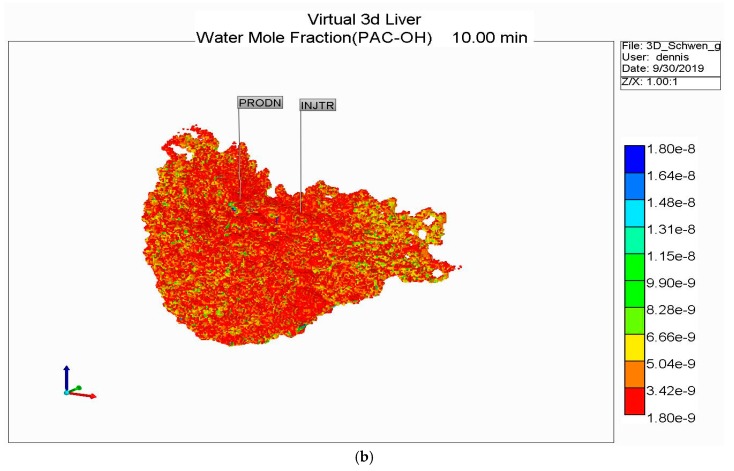
Reactive PAC and PACOH distributions in a dual continuum model (no diffusion). (**a**) Reactive PAC distributions in the fracture after 10 min (with diffusion). (**b**) Reactive PAC-OH distributions in the matrix after 10 min (with diffusion).

## Appendix A

### Full Organ Scale—Dual Continuum Flow Simulations

This work is motivated by our earlier full-liver scale simulation model, presented in White et al. [292], which will now be applied to a 3D human liver. Here, we have generated a gridded dual continuum model of the full liver, based on the liver outline and vasculature generated by Schwen et al. [293], who employed an OCC vasculature generation algorithm. The portal vein (PV) and hepatic vein (HV) vasculature provided by Schwen was grouped into five generations each based on the radius size. The root radius for PV is 3.2 mm, while the root radius for HV is 2.8 mm. It is emphasized that Schwen et al. did not extend their algorithm to further generations of smaller radii. For reference, the ultimate expected sinusoid radius is approximately 3 um (3 × 10^−3^ mm).

With a chosen grid size of 1 mm^3^, our resulting discretized model is 227 × 170 × 175 = 7,653,250 cubic grid cells, but with only 3,291,430 active grid cells. Of these cells, a fraction is occupied by Schwen vasculature, while the dominant remaining fraction of grid cells approximates liver lobules. With our chosen grid size of 1 mm, the largest generations of Schwen vasculature occupy multiple grid cells in cross section and length. The lobule grid cells are represented as a dual continuum of sinusoid vasculature and tissue. This represents the simplest gridded dual continuum representation of the full liver organ. Further improvements to this basic model include (a) upscaling liver lobule properties to account for the impact of missing fine scale vasculature and (b) adjusting the gridded Schwen vasculature to partial occupancy of a 1 mm^3^ grid cell.

With this basic model, several simulations were run. A non-reactive PAC injection case including only convective transfer between vasculature and tissue was compared with a case including an additional diffusive transfer mechanism. These two cases were then rerun with a tissue reaction, converting injected PAC to PAC-OH. Note that no consideration was paid to possible upscaling of the diffusive and reactive terms used in these models. The parameters from our lobule-scale models were used directly.

There was little observed difference in produced PAC dynamics with these runs (produced PAC reached injected levels after 2 min, and no produced PAC-OH was observed in the reactive cases up to 10 min). There was little transfer of PAC from vasculature to tissue without the addition of diffusive transfer, and this had a significant observable effect on the internal PAC distribution in the absence of a reaction, and also on the distribution of PAC-OH for the reactive cases. An interesting result of the reaction-plus-diffusion case is that PAC was found to be 100% in the vasculature (none in tissue), while PAC-OH concentrations were almost identical in vasculature and tissue continua locally.

More specifically, for the no-diffusion case, Figure A1a shows unreacted PAC in the vasculature after 10 min, while A1b shows reacted PAC-OH in the tissue at the same time. Because both the large vasculature and the lobule’s vasculature contribute to the vasculature dual continuum, we can see two distinct distributions of PAC in the vasculature (i.e., a high concentration distribution and a low concentration distribution, found in the large vasculature and sinusoidal vasculature, respectively). Figure A1b shows minimally reacted PAC-OH in the tissue at the same time point. Not shown are reacted PAC-OH in the vasculature and unreacted PAC in tissue, but both concentrations are essentially zero.

This behavior is dramatically altered in the reaction with the diffusive transfer case, as shown in Figure A2. The additional (i.e., dominant) diffusive transfer to tissue results in the lower PAC concentrations being completely removed from the vasculature (Figure A2a), resulting in large conversion to PAC-OH in tissue (Figure A2b). Not shown are the negligible amount of PAC in the tissue continua and the almost identical concentration of PAC-OH in vasculature to that in tissue for this highly reactive case (as mentioned above).
